# Taking inspiration from birds to improve flow in prosthetic heart valves: an European Research Council granted project

**DOI:** 10.1093/eurheartj/ehae330

**Published:** 2024-07-10

**Authors:** Yevgeniy Kreinin, Mark Epshtein, Gil Bolotin, Netanel Korin

**Affiliations:** Department of Biomedical Engineering, Technion Israel Institute of Technology, Technion City, Haifa 3200003, Israel; Department of Biomedical Engineering, Technion Israel Institute of Technology, Technion City, Haifa 3200003, Israel; Department of Radiology, New England Center for Stroke Research, University of Massachusetts Medical School, Worcester, MA, USA; Department of Cardiac Surgery, Rambam Health Care Campus, Haifa 3109601, Israel; Technion—IIT, The Ruth Bruce Rappaport Faculty of Medicine, Haifa 3525433, Israel; Department of Biomedical Engineering, Technion Israel Institute of Technology, Technion City, Haifa 3200003, Israel

##  

### The clinical problem

Valvular heart disease affects more than 100 million people worldwide and is associated with significant morbidity and mortality.^1^ Fortunately, prosthetic heart valves (PHV), both biological (BHV) and mechanical (MHV), have improved the survival rate and the quality of life of aortic valve heart-disease patients over the last six decades. While BHVs, taken from animal tissue and implanted via a transcatheter aortic valve replacement (TAVR) procedure, show improved haemodynamics, their durability is limited and frequently require replacement within 10–15 years.^2^ However, MHVs, surgically implanted, can last a lifetime but necessitate lifelong anticoagulation therapy to mitigate thrombosis risks. Thus, there is a critical need for durable PHVs that do not require anticoagulants.

Blood flow around an implanted valve is inherently never the same as the physiological flow, potentially promoting thrombosis. Despite advancements, surgically implanted MHVs still encounter flow-related thrombosis, particularly in stagnation zones around the annulus,^3^ see *Figure 1* upper panel. Moreover, BHVs, commonly used in transcatheter procedures, have seen increased thrombosis reports in clinical settings, ranging from 4.5% to 40% over the past decade.^1^ As a result of its structure, TAVR valves inherently produce pro-thrombotic pathological flow structures, see *Figure 1* bottom panel. Thus, despite material engineering improvements utilizing durable, low-thrombogenic materials in PHVs, and particularly in polymeric HVs,^4^ flow-associated thrombosis remains a clinical problem.

**Figure 1:**
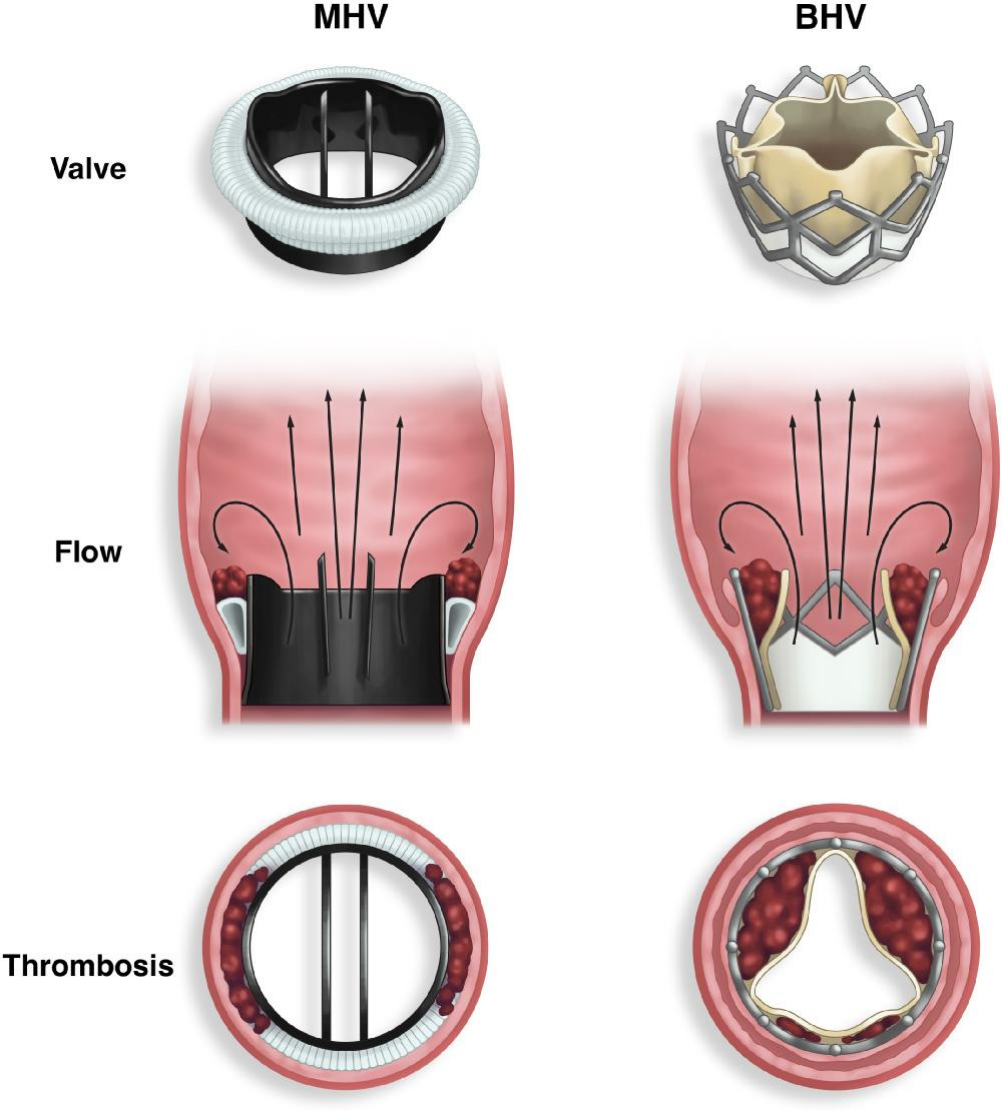
The clinical problem: PHV thrombosis. Flow-related thrombosis occurs in all prosthetic heart valves. Due to their structure and implantation method, prosthetic heart valves result in pathological flow that supports thrombotic complications. Left: Surgically implanted MHVs produce high shear and stagnation zones around the valve, where clots tend to accumulate. Right: BHVs that are implanted via a TAVR procedure produce abnormal flow, such as stagnation zones around the leaflets and stents where clots can accumulate

## ERC-funded ‘StreamlineValve Project’

The ERC-funded Proof-of-Concept (PoC) ‘StreamlineValve’ grant is an interdisciplinary project that will unite basic scientists and engineers from the Technion—Israeli Institute of Technology with clinician scientists from Israel Health Care Campus—Rambam to tackle the issue of flow-related thrombosis in PHVs. The concept for the ‘StreamlineValve Project’ emerged from our ERC-funded project (VasoSurfer, 101002057), which focuses on a novel strategy for treating aneurysms using injectable biomaterials and surface tension phenomena. Notably, saccular aneurysms frequently exhibit flow-related thrombus formation, similar to the clot accumulation observed on heart valves. Therefore, designing PHVs that circumvent pathological flow structures, which promote thrombosis, can be valuable in addressing this challenge. Furthermore, our approach draws inspiration from flow control mechanisms observed in nature (e.g. whale swimming and bird flying) to enhance performance.

### Taking inspiration from nature

**Figure 2:**
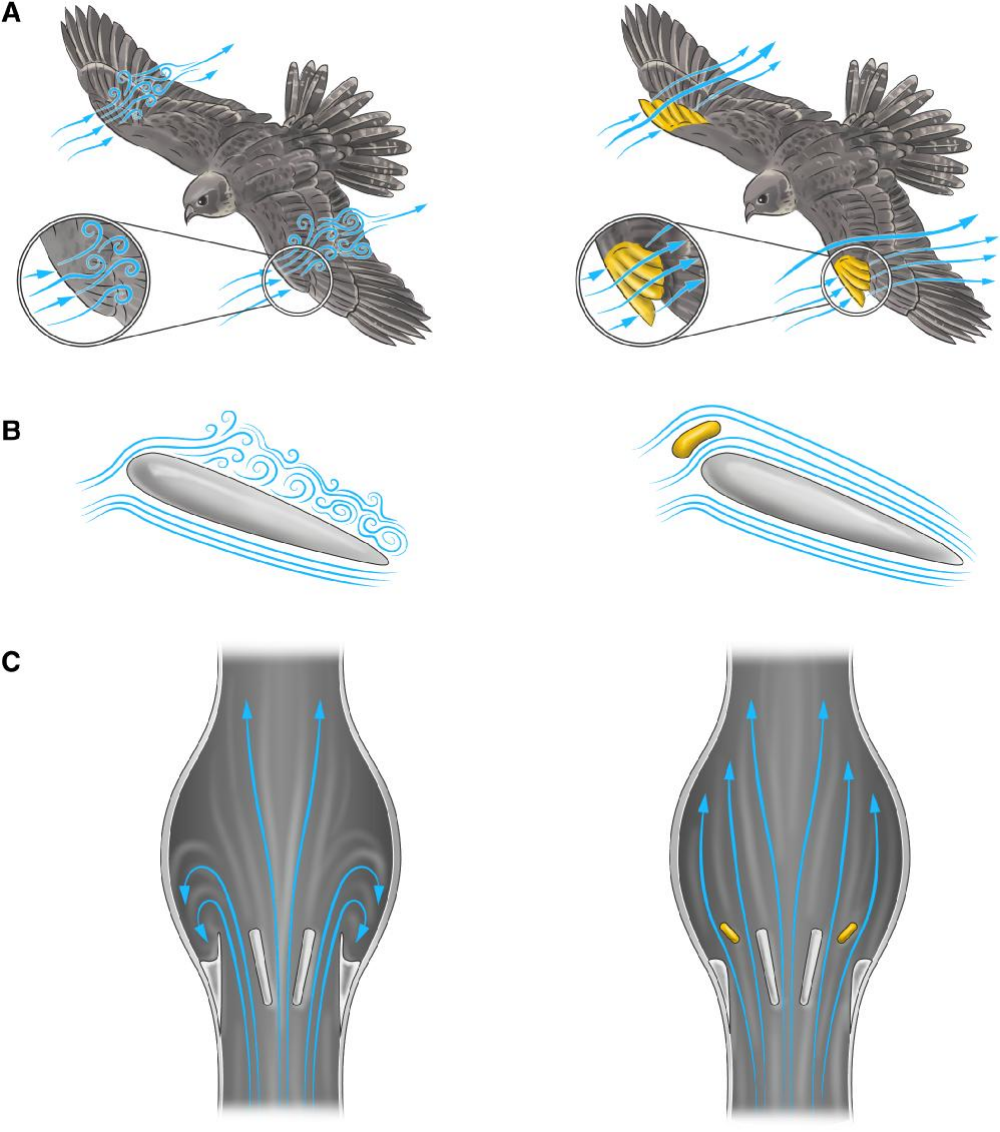
A passive flow-controlled valve to inhibit PHV thrombosis and improve valve performance (StreamlineValve). (*A*) Flow control is used in nature for flight optimization. Schematic showing the use of the alula, a small structure in the wings of birds, which can increase lift and delay stall. (*B*) In the aerodynamic industry, flow control approaches have improved aircraft design. Schematic showing the use of a slotted aerofoil to reduce air flow separation (*C*) In a regular valve, abnormal flow and stagnation zones are prone to thrombosis (left). In the flow-controlled valve, a small volume of the blood flow is redirected to reduce stagnation zones as well as modulate the flow profile to be less turbulent and overall reducing flow-related thrombosis (right)

Flow Control is a field of fluid dynamics that refers to manipulating and managing fluid flow via a small configuration change to provide a considerable engineering benefit (e.g. drag reduction, lift increase, and mixing enhancement). In nature, passive flow control, which does not involve external energy, is also used to optimize swimming and flying, amongst other applications (for example: some birds use passively pop-up flaps to enhance the lift force via reducing flow separations, see *Figure 2A*). Flow control has also been widely employed in aircraft engineering to optimize and stabilize flight while minimizing flow disruptions caused by turbulence and separated airflow, see *Figure 2B,* where passive flow control has revolutionized aviation by replacing traditional moving aerodynamic surfaces with more advanced techniques.^5^ Although there has been some work trying to apply this general concept to improve cardiovascular devices, such as using vortex generators in mechanical heart valves,^6^ its general application in cardiovascular devices and in PHVs so far has been very limited.

### The StreamlineValve project—a flow-controlled valve

In the StreamlineValve project, we plan to use passive flow control strategies to modulate the flow field such that it reduces primary factors contributing to coagulation on PHV, for instance, reducing stagnation areas, residence time, high shear stresses, and turbulence,^7^ see *Figure 2C*. The redirection of flow allows the flow-controlled PHV to act as a self-cleaning valve that maintains a clot free environment around the valve. Moreover, using this approach could also lead to a homogenous pressure across all flow regions, consequently improving valve fluctuations, reducing the peak bloodstream velocity and hence reducing turbulence zones, and uniquely ensuring uninterrupted blood flow to coronary arteries through the valve’s distinctive structure. We have already demonstrated this concept successfully *in vitro* in a modified MHV, where our StreamlineValve MHV resulted in a significant reduction in clot accumulation compared to a regular MHV. In this PoC project, we propose to extend this to the future of prosthetic HVs via a flow-controlled polymeric HV that can be implanted in a simple TAVR procedure, avoiding undesired surgical procedures. Ultimately, this new approach may improve valve durability and patient safety, which is particularly crucial for young patients facing lifelong implantation and associated risks.
